# *N*,*N*′-[1,4-Phenyl­enebis(methyl­ene)]bis­(*N*,*N*-diethyl­ethanaminium) dibromide

**DOI:** 10.1107/S1600536812000141

**Published:** 2012-01-11

**Authors:** Munirah Sufiyah Abdul Rahim, Hamid Khaledi, Yatimah Alias, Urs Welz-Biermann

**Affiliations:** a University of Malaya Centre for Ionic Liquids, Department of Chemistry, University of Malaya, 50603 Kuala Lumpur, Malaysia; b Department of Chemistry, University of Malaya, 50603 Kuala Lumpur, Malaysia; c China Ionic Liquid Laboratory, Dalian Institute of Chemical Physics, Chinese, Academy of Sciences, 116023 Dalian, People’s Republic of China

## Abstract

In the crystal structure of the title compound, C_20_H_38_N_2_^2+^·2Br^−^, the centroid of the aromatic ring is located on an inversion center, so that the asymmetric unit consists of one-half mol­ecule of the dication and one bromide anion. C—H⋯Br inter­actions connect the two components into a three-dimensional network. An intra­molecular C—H⋯π inter­action is also observed.

## Related literature

For the properties of dicationic ionic liquids, see: Anderson *et al.* (2005 ▶). For the structure of *p*-phenyl­enedimethanaminium dibromide, see: Zhang & Han (2010 ▶).
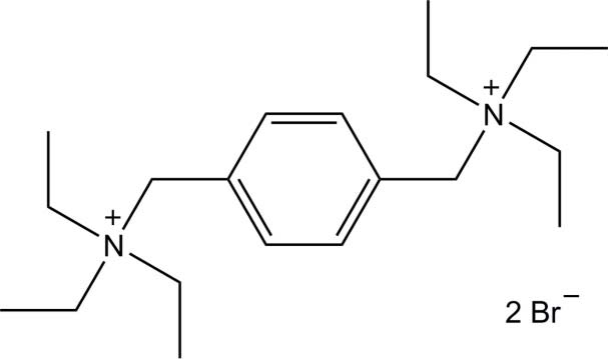


## Experimental

### 

#### Crystal data

C_20_H_38_N_2_^2+^·2Br^−^
*M* *_r_* = 466.34
Monoclinic, 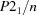

*a* = 8.2713 (5) Å
*b* = 14.1440 (9) Å
*c* = 9.0762 (6) Å
β = 97.634 (1)°
*V* = 1052.41 (12) Å^3^
*Z* = 2
Mo *K*α radiation
μ = 3.86 mm^−1^
*T* = 100 K
0.51 × 0.47 × 0.35 mm

#### Data collection

Bruker APEXII CCD diffractometer
Absorption correction: multi-scan (*SADABS*; Sheldrick, 1996 ▶) *T*_min_ = 0.244, *T*_max_ = 0.346
10066 measured reflections
2304 independent reflections
2093 reflections with *I* > 2σ(*I*)
*R*_int_ = 0.021

#### Refinement

*R*[*F* ^2^ > 2σ(*F* ^2^)] = 0.017
*wR*(*F* ^2^) = 0.043
*S* = 1.06
2304 reflections
112 parameters
H-atom parameters constrained
Δρ_max_ = 0.43 e Å^−3^
Δρ_min_ = −0.23 e Å^−3^

### 

Data collection: *APEX2* (Bruker, 2007 ▶); cell refinement: *SAINT* (Bruker, 2007 ▶); data reduction: *SAINT*; program(s) used to solve structure: *SHELXS97* (Sheldrick, 2008 ▶); program(s) used to refine structure: *SHELXL97* (Sheldrick, 2008 ▶); molecular graphics: *X-SEED* (Barbour, 2001 ▶); software used to prepare material for publication: *SHELXL97* and *publCIF* (Westrip, 2010 ▶).

## Supplementary Material

Crystal structure: contains datablock(s) I, global. DOI: 10.1107/S1600536812000141/is5042sup1.cif

Structure factors: contains datablock(s) I. DOI: 10.1107/S1600536812000141/is5042Isup2.hkl

Supplementary material file. DOI: 10.1107/S1600536812000141/is5042Isup3.cml

Additional supplementary materials:  crystallographic information; 3D view; checkCIF report

## Figures and Tables

**Table 1:** Hydrogen-bond geometry (Å, °) *Cg* is the centroid of the aromatic ring.

*D*—H⋯*A*	*D*—H	H⋯*A*	*D*⋯*A*	*D*—H⋯*A*
C7—H7*A*⋯Br1	0.99	2.80	3.7565 (14)	163
C2—H2*B*⋯Br1^i^	0.99	2.90	3.7716 (14)	148
C6—H6*B*⋯Br1^ii^	0.99	2.92	3.8318 (14)	153
C7—H7*B*⋯Br1^ii^	0.99	2.89	3.7832 (14)	150
C1—H1*C*⋯*Cg*	0.98	2.74	3.6529 (16)	156

Symmetry codes: (i) 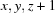; (ii) 
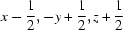
.

**Fig. 1.**
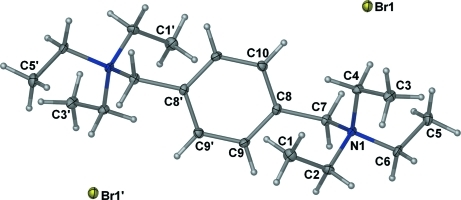
Molecular structure of the title compound with displacement ellipsoids drawn at 50% probability level. Hydrogen atoms are drawn as spheres of arbitrary radius. [Symmetry code: ' = -x + 1, -y + 1, -z + 1.]

## supplementary crystallographic information

### Comment

In comparison to the common singly charged ionic liquids, dicationic ionic liquids are a new class of organic salts having two positive charges on the same cation. They have been proved to have a better performance in terms of thermal stability, wide liquidus range, and unusual dissolution properties (Anderson *et al.*, 2005). The title compound was synthesized as a precursor for the synthesis of dicationic ionic liquids with a rigid phenylene group spacer.

Similar to the structure of *p*-phenylenedimethanaminium dibromide (Zhang & Han, 2010), the centroid of the aromatic ring is located on an inversion center, therefore the asymmetric unit consists of one-half of the dication and one bromide anion. In the crystal, the cations and anions are linked into a three-dimensional polymeric structure *via* C—H···Br interactions. The network is further condolidated by intramolecular C—H···*π* interactions (Table 1).

### Experimental

Triethylamine (3.06 ml, 22 mmol) was added dropwise to a solution of α,α-dibromo-*p*-xylene (2.64 g, 10 mmol) in acetonitrile (50 ml). The mixture was stirred at room temperature for 24 hr. The white precipitate of the product was filtered and washed with acetonitrile and dried under vacuum. Recrystallization of the dicationic salt from methanol afforded crystals suitable for X-ray crystallographicanalysis

### Refinement

H atoms were placed at calculated positions and refined as riding atoms, with C—H distances of 0.95 (aryl), 0.98 (methyl) and 0.99 (methylene) Å, and with *U*_iso_(H) set to 1.2 (1.5 for methyl) *U*_eq_(C).

### Crystal data

**Table:**

C_20_H_38_N_2_^2+^·2Br^−^	*F*(000) = 484
*M**_r_* = 466.34	*D*_x_ = 1.472 Mg m^−^^3^
Monoclinic, *P*2_1_/*n*	Mo *K*α radiation, λ = 0.71073 Å
Hall symbol: -P 2yn	Cell parameters from 8155 reflections
*a* = 8.2713 (5) Å	θ = 2.7–28.3°
*b* = 14.1440 (9) Å	µ = 3.86 mm^−^^1^
*c* = 9.0762 (6) Å	*T* = 100 K
β = 97.634 (1)°	Block, colorless
*V* = 1052.41 (12) Å^3^	0.51 × 0.47 × 0.35 mm
*Z* = 2	

### Data collection

**Table:**

Bruker APEXII CCD diffractometer	2304 independent reflections
Radiation source: fine-focus sealed tube	2093 reflections with *I* > 2σ(*I*)
graphite	*R*_int_ = 0.021
φ and ω scans	θ_max_ = 27.0°, θ_min_ = 2.7°
Absorption correction: multi-scan (*SADABS*; Sheldrick, 1996)	*h* = −10→10
*T*_min_ = 0.244, *T*_max_ = 0.346	*k* = −16→18
10066 measured reflections	*l* = −11→11

### Refinement

**Table:**

Refinement on *F*^2^	Primary atom site location: structure-invariant direct methods
Least-squares matrix: full	Secondary atom site location: difference Fourier map
*R*[*F*^2^ > 2σ(*F*^2^)] = 0.017	Hydrogen site location: inferred from neighbouring sites
*wR*(*F*^2^) = 0.043	H-atom parameters constrained
*S* = 1.06	*w* = 1/[σ^2^(*F*_o_^2^) + (0.019*P*)^2^ + 0.5002*P*] where *P* = (*F*_o_^2^ + 2*F*_c_^2^)/3
2304 reflections	(Δ/σ)_max_ = 0.001
112 parameters	Δρ_max_ = 0.43 e Å^−^^3^
0 restraints	Δρ_min_ = −0.23 e Å^−^^3^

### Special details

**Table:**

Geometry. All e.s.d.'s (except the e.s.d. in the dihedral angle between two l.s. planes) are estimated using the full covariance matrix. The cell e.s.d.'s are taken into account individually in the estimation of e.s.d.'s in distances, angles and torsion angles; correlations between e.s.d.'s in cell parameters are only used when they are defined by crystal symmetry. An approximate (isotropic) treatment of cell e.s.d.'s is used for estimating e.s.d.'s involving l.s. planes.
Refinement. Refinement of *F*^2^ against ALL reflections. The weighted *R*-factor *wR* and goodness of fit *S* are based on *F*^2^, conventional *R*-factors *R* are based on *F*, with *F* set to zero for negative *F*^2^. The threshold expression of *F*^2^ > σ(*F*^2^) is used only for calculating *R*-factors(gt) *etc*. and is not relevant to the choice of reflections for refinement. *R*-factors based on *F*^2^ are statistically about twice as large as those based on *F*, and *R*- factors based on ALL data will be even larger.

### Fractional atomic coordinates and isotropic or equivalent isotropic displacement parameters (Å^2^)

**Table:**

	*x*	*y*	*z*	*U*_iso_*/*U*_eq_	
N1	0.82236 (14)	0.33100 (8)	0.70235 (13)	0.0099 (2)	
C1	0.72184 (18)	0.46030 (11)	0.87038 (16)	0.0176 (3)	
H1A	0.8299	0.4897	0.8894	0.026*	
H1B	0.6651	0.4681	0.9578	0.026*	
H1C	0.6585	0.4906	0.7843	0.026*	
C2	0.74049 (18)	0.35590 (10)	0.83906 (15)	0.0138 (3)	
H2A	0.6308	0.3267	0.8271	0.017*	
H2B	0.8045	0.3266	0.9273	0.017*	
C3	1.10397 (18)	0.38839 (11)	0.80909 (16)	0.0156 (3)	
H3A	1.0615	0.3870	0.9049	0.023*	
H3B	1.1769	0.4428	0.8062	0.023*	
H3C	1.1644	0.3300	0.7967	0.023*	
C4	0.96263 (16)	0.39682 (10)	0.68396 (15)	0.0120 (3)	
H4A	0.9223	0.4628	0.6796	0.014*	
H4B	1.0028	0.3828	0.5883	0.014*	
C5	0.97084 (18)	0.18930 (11)	0.60528 (17)	0.0166 (3)	
H5A	0.8954	0.1845	0.5127	0.025*	
H5B	1.0132	0.1264	0.6346	0.025*	
H5C	1.0616	0.2311	0.5900	0.025*	
C6	0.88154 (17)	0.22941 (10)	0.72681 (16)	0.0131 (3)	
H6A	0.9550	0.2263	0.8221	0.016*	
H6B	0.7862	0.1887	0.7364	0.016*	
C7	0.69822 (17)	0.33096 (10)	0.56112 (15)	0.0113 (3)	
H7A	0.7577	0.3197	0.4750	0.014*	
H7B	0.6231	0.2770	0.5668	0.014*	
C8	0.59739 (16)	0.41916 (10)	0.53110 (15)	0.0110 (3)	
C9	0.43874 (17)	0.42236 (10)	0.56832 (15)	0.0124 (3)	
H9	0.3958	0.3692	0.6139	0.015*	
C10	0.65646 (17)	0.49722 (10)	0.46095 (15)	0.0126 (3)	
H10	0.7630	0.4954	0.4329	0.015*	
Br1	0.937757 (16)	0.344754 (10)	0.237945 (15)	0.01420 (6)	

### Atomic displacement parameters (Å^2^)

**Table:**

	*U*^11^	*U*^22^	*U*^33^	*U*^12^	*U*^13^	*U*^23^
N1	0.0107 (5)	0.0084 (6)	0.0103 (5)	0.0003 (5)	−0.0002 (4)	0.0017 (4)
C1	0.0219 (7)	0.0177 (8)	0.0136 (7)	0.0005 (6)	0.0034 (6)	−0.0040 (6)
C2	0.0152 (7)	0.0164 (7)	0.0102 (6)	−0.0006 (6)	0.0035 (5)	−0.0001 (5)
C3	0.0136 (7)	0.0160 (8)	0.0162 (7)	−0.0026 (6)	−0.0022 (5)	0.0011 (6)
C4	0.0126 (6)	0.0107 (7)	0.0126 (6)	−0.0029 (5)	0.0009 (5)	0.0008 (5)
C5	0.0162 (7)	0.0118 (7)	0.0212 (7)	0.0033 (6)	0.0005 (6)	−0.0012 (6)
C6	0.0137 (6)	0.0089 (7)	0.0160 (7)	0.0011 (6)	−0.0007 (5)	0.0034 (6)
C7	0.0111 (6)	0.0112 (7)	0.0108 (6)	−0.0007 (5)	−0.0020 (5)	−0.0008 (5)
C8	0.0122 (6)	0.0105 (7)	0.0095 (6)	0.0009 (5)	−0.0017 (5)	−0.0005 (5)
C9	0.0132 (6)	0.0115 (7)	0.0124 (6)	−0.0013 (5)	0.0011 (5)	0.0029 (5)
C10	0.0106 (6)	0.0149 (7)	0.0121 (6)	0.0001 (5)	0.0012 (5)	0.0006 (5)
Br1	0.01526 (8)	0.01314 (8)	0.01435 (8)	0.00247 (5)	0.00246 (5)	0.00042 (5)

### Geometric parameters (Å, °)

**Table:**

N1—C4	1.5140 (17)	C5—C6	1.517 (2)
N1—C6	1.5246 (18)	C5—H5A	0.9800
N1—C2	1.5314 (17)	C5—H5B	0.9800
N1—C7	1.5322 (17)	C5—H5C	0.9800
C1—C2	1.516 (2)	C6—H6A	0.9900
C1—H1A	0.9800	C6—H6B	0.9900
C1—H1B	0.9800	C7—C8	1.5052 (19)
C1—H1C	0.9800	C7—H7A	0.9900
C2—H2A	0.9900	C7—H7B	0.9900
C2—H2B	0.9900	C8—C10	1.395 (2)
C3—C4	1.5225 (19)	C8—C9	1.3986 (19)
C3—H3A	0.9800	C9—C10^i^	1.389 (2)
C3—H3B	0.9800	C9—H9	0.9500
C3—H3C	0.9800	C10—C9^i^	1.389 (2)
C4—H4A	0.9900	C10—H10	0.9500
C4—H4B	0.9900		
			
C4—N1—C6	111.08 (10)	H4A—C4—H4B	107.8
C4—N1—C2	112.07 (11)	C6—C5—H5A	109.5
C6—N1—C2	105.47 (10)	C6—C5—H5B	109.5
C4—N1—C7	110.25 (10)	H5A—C5—H5B	109.5
C6—N1—C7	106.74 (10)	C6—C5—H5C	109.5
C2—N1—C7	111.01 (10)	H5A—C5—H5C	109.5
C2—C1—H1A	109.5	H5B—C5—H5C	109.5
C2—C1—H1B	109.5	C5—C6—N1	115.13 (12)
H1A—C1—H1B	109.5	C5—C6—H6A	108.5
C2—C1—H1C	109.5	N1—C6—H6A	108.5
H1A—C1—H1C	109.5	C5—C6—H6B	108.5
H1B—C1—H1C	109.5	N1—C6—H6B	108.5
C1—C2—N1	116.30 (12)	H6A—C6—H6B	107.5
C1—C2—H2A	108.2	C8—C7—N1	116.41 (11)
N1—C2—H2A	108.2	C8—C7—H7A	108.2
C1—C2—H2B	108.2	N1—C7—H7A	108.2
N1—C2—H2B	108.2	C8—C7—H7B	108.2
H2A—C2—H2B	107.4	N1—C7—H7B	108.2
C4—C3—H3A	109.5	H7A—C7—H7B	107.3
C4—C3—H3B	109.5	C10—C8—C9	118.77 (13)
H3A—C3—H3B	109.5	C10—C8—C7	121.29 (12)
C4—C3—H3C	109.5	C9—C8—C7	119.88 (13)
H3A—C3—H3C	109.5	C10^i^—C9—C8	120.44 (13)
H3B—C3—H3C	109.5	C10^i^—C9—H9	119.8
N1—C4—C3	113.17 (11)	C8—C9—H9	119.8
N1—C4—H4A	108.9	C9^i^—C10—C8	120.76 (13)
C3—C4—H4A	108.9	C9^i^—C10—H10	119.6
N1—C4—H4B	108.9	C8—C10—H10	119.6
C3—C4—H4B	108.9		

Symmetry codes: (i) −*x*+1, −*y*+1, −*z*+1.

### Hydrogen-bond geometry (Å, °)

**Table:**

*Cg* is the centroid of the aromatic ring.

**Table:**

*D*—H···*A*	*D*—H	H···*A*	*D*···*A*	*D*—H···*A*
C7—H7A···Br1	0.99	2.80	3.7565 (14)	163.
C2—H2B···Br1^ii^	0.99	2.90	3.7716 (14)	148.
C6—H6B···Br1^iii^	0.99	2.92	3.8318 (14)	153.
C7—H7B···Br1^iii^	0.99	2.89	3.7832 (14)	150.
C1—H1C···Cg	0.98	2.74	3.6529 (16)	156

Symmetry codes: (ii) *x*, *y*, *z*+1; (iii) *x*−1/2, −*y*+1/2, *z*+1/2.
